# The Microstructure and Electronic Properties of Yttrium Oxide Doped With Cerium: A Theoretical Insight

**DOI:** 10.3389/fchem.2020.00338

**Published:** 2020-04-28

**Authors:** Meng Ju, Jingjing Wang, Jing Huang, Chuanzhao Zhang, Yuanyuan Jin, Weiguo Sun, Shichang Li, Yunhong Chen

**Affiliations:** ^1^School of Physical Science and Technology, Southwest University, Chongqing, China; ^2^College of Computer and Information Engineering, Hubei Normal University, Huangshi, China; ^3^Department of Physics and Optoelectronic Engineering, Yangtze University, Jingzhou, China; ^4^Centre for Science at Extreme Conditions and School of Physics and Astronomy, Scottish Universities Physics Alliance (SUPA), University of Edinburgh, Edinburgh, United Kingdom; ^5^School of Science, Chongqing University of Posts and Telecommunications, Chongqing, China

**Keywords:** crystal structure prediction (CSP), electronic propercies, structural evolution, rare earth element, theoretical calculation DFT

## Abstract

Trivalent Cerium (Ce^3+^) doped Yttrium Oxide (Y_2_O_3_) host crystal has drawn considerable interest due to its popular optical 5d-4f transition. The outstanding optical properties of Y_2_O_3_:Ce system have been demonstrated by previous studies but the microstructures still remain unclear. The lacks of Y_2_O_3_:Ce microstructures could constitute a problem to further exploit its potential applications. In this sense, we have comprehensively investigated the structural evolutions of Y_2_O_3_:Ce crystals based on the CALYPSO structure search method in conjunction with density functional theory calculations. Our result uncovers a new rhombohedral phase of Y_2_O_3_:Ce with R-3 group symmetry. In the host crystal, the Y^3+^ ion at central site can be naturally replaced by the doped Ce^3+^, resulting in a perfect cage-like configuration. We find an interesting phase transition that the crystallographic symmetry of Y_2_O_3_ changes from cubic to rhombohedral when the impurity Ce^3+^ is doped into the host crystal. With the nominal concentration of Ce^3+^ at 3.125%, many metastable structures are also identified due to the different occupying points in the host crystal. The X-ray diffraction patterns of Y_2_O_3_:Ce are simulated and the theoretical result is comparable to experimental data, thus demonstrating the validity of the lowest energy structure. The result of phonon dispersions shows that the ground state structure is dynamically stable. The analysis of electronic properties indicate that the Y_2_O_3_:Ce possesses a band gap of 4.20 eV which suggests that the incorporation of impurity Ce^3+^ ion into Y_2_O_3_ host crystal leads to an insulator to semiconductor transition. Meanwhile, the strong covalent bonds of O atoms in the crystal, which may greatly contribute to the stability of ground state structure, are evidenced by electron localization function. These obtained results elucidate the structural and bonding characters of Y_2_O_3_:Ce and could also provide useful insights for understanding the experimental phenomena.

## Introduction

The rare-earth Cerium ions doped crystals constitute an attractive class of materials that have been extensively used in many kinds of fields including scintillation phosphors, laser medium, and white light emitting diode phosphors (Han et al., 2019; Lin et al., 2019; Masanori et al., 2020). The outstanding optical behaviors of trivalent Cerium ion (Ce^3+^) has drawn considerable interest due to its popular optical 5d-4f transition. Among various host materials, Yttrium Oxide (Y_2_O_3_) crystal is considered to be the most promising sesquioxide host because of its unique chemical and thermal stability. The Y_2_O_3_ host crystal is also one of the multifunctional materials that can give rise to many application areas owing to its fabulous capacity of incorporating the activated laser ions (Ming et al., 2018; Wang et al., 2018; Ju et al., 2020). The latest study has indicated that the Ce^3+^ doped Y_2_O_3_ crystals (Y_2_O_3_:Ce) exhibit dominant emission bands at around 380 nm and relatively low intensive band at 560 nm (Gieszczyk et al., 2019). The results further demonstrate the ideal applications of energy storage phosphors for Y_2_O_3_:Ce. The excellent advantages of Y_2_O_3_:Ce can also be evidenced by the effective use as various laser ceramics (Lupei et al., 2017).

It is well-known that the laser actions can be generally identified in the absorption and emission spectra of rare-earth doped materials. In order to explore the luminescent properties of Y_2_O_3_:Ce, Jia et al. (2001) had synthesized the Y_2_O_3_:Ce nanoparticles in experiments and measured the photoluminescence spectra at room temperature. Their results revealed that the strong emissions cover the ultraviolet band from 240 nm to 380 nm. To explain the emission lines of the spectra, Loitongbam et al. (2013) measured the luminescence intensities of Y_2_O_3_:Ce and found that the characteristic blue color emissions at 424 and 486 nm are originated from Ce^3+^ ion 5d (spectra terms) → 4f (spectra terms). An unexpected optical activity, including up and down conversions, for Y_2_O_3_:Ce crystal was firstly observed by Marin et al. (2013). Although the laser actions were established by a few studies, many researchers were motivated to probe the structural properties of Y_2_O_3_:Ce. The effect of doping Ce^3+^ ion into Y_2_O_3_ fibers was investigated by Zhu et al. (2008). They found that the obvious quenching of the luminescence occurred at Ce^3+^ concentration of 5%. By using the solid-state-reactive method, Liu et al. (2020) carried out a study on the structures of a series of Ce^3+^ doped Y_2_O_3_ ternary ceramics. The results demonstrated that the solubility of Ce^3+^ concentration at 4% could broaden emission spectra and lead to a large red-shift, which is attractive for the white light emitting. A recent research on the structural properties of Y_2_O_3_:Ce was conducted by Krutikova et al. (2020). The nanopowders were obtained by laser ablation and the X-ray diffraction (XRD) patterns of Y_2_O_3_:Ce crystal were reported. By looking at the investigations concerning Y_2_O_3_:Ce in the literatures, it can be concluded that the systematic electronic structures have not yet been explored, especially for the theoretical insights. Furthermore, the lacks of Y_2_O_3_:Ce microstructures constitute a problem to exploit its potential prospects in many applications.

In this paper, we have performed a systematic study on the stable structures and electronic properties for Y_2_O_3_ doped with Ce^3+^ system. By using the CALYPSO (Crystal structure AnaLYsis by Particle Swarm Optimization) structure search method (Wang et al., 2010, 2012; Li et al., 2014) combined with first-principle calculation, the low-lying energy structures of Y_2_O_3_:Ce are extensively searched. A large number of candidate structures are obtained and the ground state structure together with the first four metastable structures is analyzed in detail. Based on the obtained lowest energy structure of Y_2_O_3_:Ce, we thoroughly conduct a calculation of the electronic properties, which could provide powerful guidance for further experimental and theoretical studies.

## Computational Details

We have carried out an unbiased structure search for Y_2_O_3_ doped with Ce^3+^ system based on the CALYPSO method (Wang et al., 2010, 2012; Li et al., 2014). The CALYPSO is able to successfully predict the stable structures only with given chemical composition of the system (Lu et al., 2013, 2017, 2018; Lu and Chen, 2018). The detailed method of CALYPSO has been reported in many papers (Ju et al., 2016, 2017, 2019a,b). In this work, the structure searches are performed for Y_2_O_3_ doped with Ce system at 80 atoms in one unit cell. The obtained low-lying energy structures are used to perform further geometric optimizations. We conduct the *ab initio* structural relaxations and electronic properties calculations in the framework of density functional theory (DFT) by using the local density approximation (LDA) exchange correlation functional, as implemented in the Vienna Ab Initio Simulation Package (VASP) (Kresse and Hafner, 1993; Kresse and Furthmuller, 1996; Perdew et al., 1996). Considering the strong f-electrons correlations within the heavy Ce^3+^ ion, an onsite Coulomb repulsion *U* = 5.0 eV is employed in the calculations (Herbst and Waston, 1978). We use the projector-augmented wave method to simulate the valence electron space of Ce, Y, and O atoms. The used electrons are 4f^1^5s^2^5p^6^5d^1^6s^2^, 4s^2^4p^6^4d^1^5s^2^, and 2s^2^2p^4^, respectively. Sufficiently fine Monkhorst-Pack k meshes and 500 eV cutoff energy have been chosen to make sure that the calculated enthalpy of each atom is <1 meV. By using a super cell approach, the phonon dispersion spectra are calculated in PHONOPY code (Atsushi et al., 1993). The electron localization function (ELF) (Becke and Edgecombe, 1990; Savin et al., 1992) analysis of Y_2_O_3_:Ce is performed and the results are depicted in the VESTA software (Momma and Izumi, 2011). The projected Crystal Orbital Hamilton Population (COHP) (Richard and Peter, 1993) are calculated by the LOBSTER code (Volker et al., 2011; Stefan et al., 2016).

## Results and Discussions

### Crystal Structures

**Figure 1:**
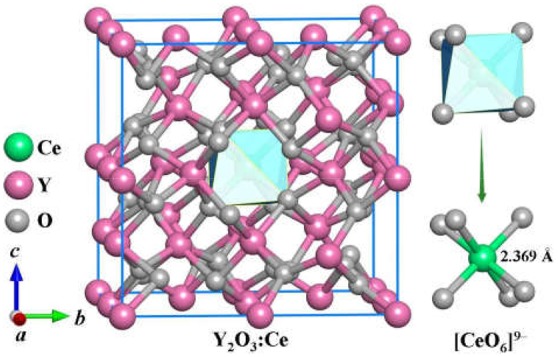
The ground state structure of Y_2_O_3_:Ce.

The stable structures for Y_2_O_3_:Ce system are favorably identified by using the method described in section Computational Details. On the basis of total energies from low to high, we have plotted the lowest-energy structure of Y_2_O_3_:Ce in Figure 1, together with the local [CeO_6_]^9−^ complex ligand. Noticeably, the ground state structure of Y_2_O_3_:Ce possesses a novel structure with R-3 (No. 148) space group. To the best of our knowledge, the rhombohedral phase of Y_2_O_3_:Ce crystal is uncovered for the first time. This result indicates an interesting phase transition that the crystallographic symmetry of Y_2_O_3_ changes from cubic (Ia-3) to rhombohedral (R-3) when the impurity Ce^3+^ is doped into the host crystal. It is clearly seen from Figure 1 that the host Y^3+^ ion can be naturally occupied by the impurity Ce^3+^ ion. Interestingly, the Wyckoff position of Ce^3+^ is 1b (0.5, 0.5, 0.5), suggesting that the ground state Y_2_O_3_:Ce is a standard cage-like structure. This result is different from that of Y_2_O_3_:Nd system (Ju et al., 2020). For reference, the coordinates of all atoms for the ground state Y_2_O_3_:Ce are summarized in Table 1. The estimated unit cell parameters and volume for Y_2_O_3_:Ce are a = b = c = 10.541 Å and 1171.371 Å (Han et al., 2019), respectively. These values are slightly smaller than those of pure Y_2_O_3_ but are comparable to the results reported by Kumar et al. (2017). As regard to the local structure, the Ce^3+^ ion is calculated to be 6-fold coordinated by O^2−^, forming the [CeO_6_]^9−^ complex ligand. The cationic site symmetry of Ce^3+^ is C_3i_ with six equal Ce–O bonds of 2.369 Å. This bond length is similar with that of Y–O bonds because the effective radius of Ce^3+^ (1.03 Å) is very close to Y^3+^ (0.90 Å).

**Table 1:** Coordinates of all atoms for the ground state Y_2_O_3_:Ce.

**Atom**	***x***	***y***	***z***	**Wyckoff site symmetry**
Ce	0.50000	0.50000	0.50000	1b
Y1	0.50000	0	0	3d
Y4	0	0.50000	0.50000	3e
Y5	0	0	0	1a
Y8	1.21723	−0.00048	−0.24957	6f
Y9	0.78317	−0.50109	−0.24904	6f
Y10	0.28219	0.00104	0.25009	6f
Y11	0.72090	−0.50141	0.24728	6f
O1	0.64138	−0.12945	−0.09800	6f
O2	1.14358	−0.62871	0.40217	6f
O3	0.35267	−0.63253	0.60591	6f
O4	0.85797	−0.12919	0.09753	6f
O5	0.85966	0.12874	0.40167	6f
O6	0.35814	−0.37026	−0.09599	6f
O7	1.14102	−0.37007	0.09751	6f
O8	0.64188	0.12739	0.59868	6f

**Figure 2:**
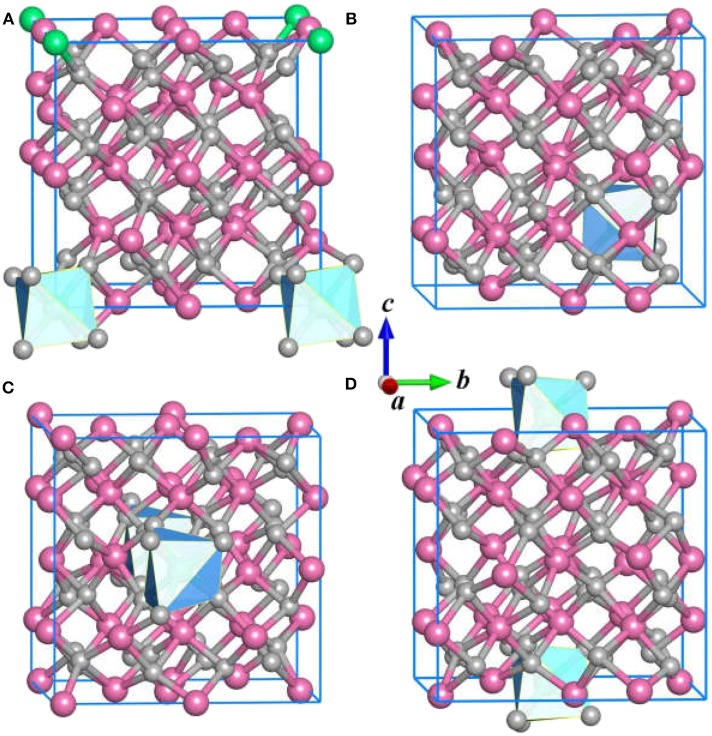
The **(A–D)** isomers for Y_2_O_3_:Ce.

In the structure prediction, we adopt the chemical composition of Ce:Y: O = 1: 31: 48 to obtain the stable structures with nominal concentration of Ce^3+^. In this sense, the impurity Ce^3+^ in Y_2_O_3_ crystal is equal to 3.125 at %. Apart from the ground state structure, the CALYPSO also identifies a large number of candidate isomers that can be useful to study the structural evolution of the Y_2_O_3_:Ce. Figure 2 illustrates the first four metastable structures of Y_2_O_3_:Ce. The isomer (a) has the same R-3 space group as the lowest energy structure while the impurity Ce^3+^ ions are likely to substitute the Y^3+^ at the lattice vertexes. The Ce^3+^ ion of isomer (a) takes the 1a (0, 0, 0) position. It is evidenced that the calculated crystal lattice parameters (10.543 Å) are nearly same as those of lowest-energy structure. The group symmetry of isomer (b) is predicted to be P1 with a triclinic phase. The Wyckoff position of Ce^3+^ is predicted to be 1a (0.25, 0.75, 0.25). Calculated result reveals that the isomer (c) exhibits a monoclinic structure which belongs to P2 symmetry. The impurity Ce^3+^ ion occupies the 1b (0, 0.47157, 05) position. For the configuration of isomer (d), it is seen that the Ce^3+^ ions appear at the center sites of bottom and top in the crystal lattice. The isomer (d) is assigned to P1 group symmetry and is 0.27 eV energetically higher than ground state structure.

**Figure 3:**
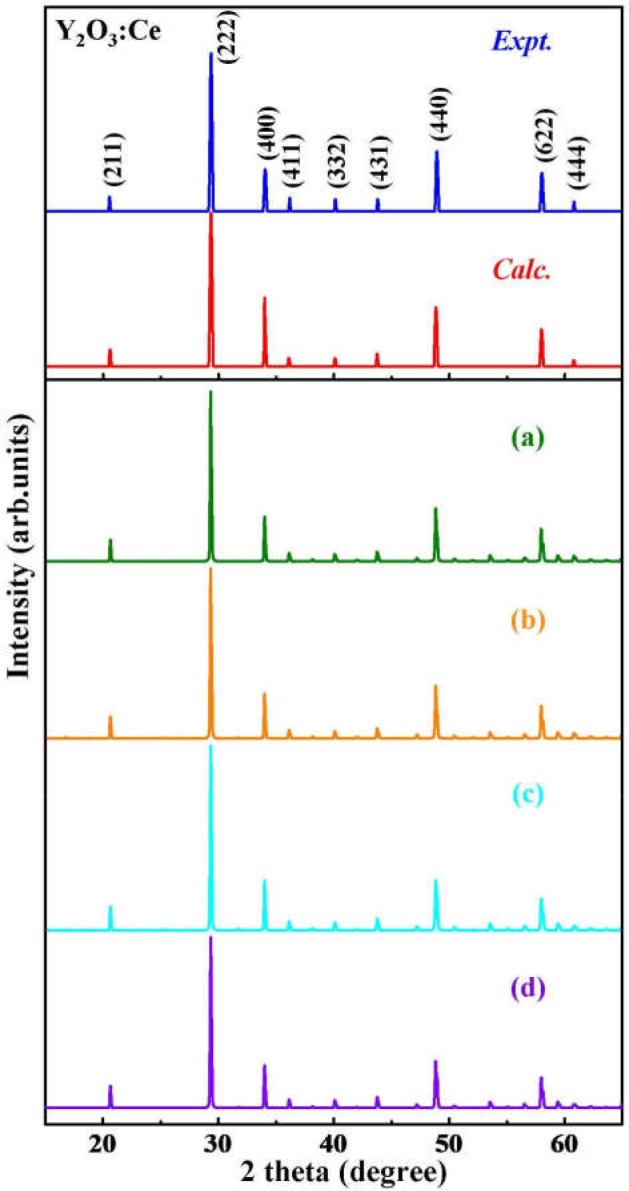
The simulated XRD patterns for ground state Y_2_O_3_:Ce and isomers **(a–d)**, compared with measured spectra.

**Figure 4:**
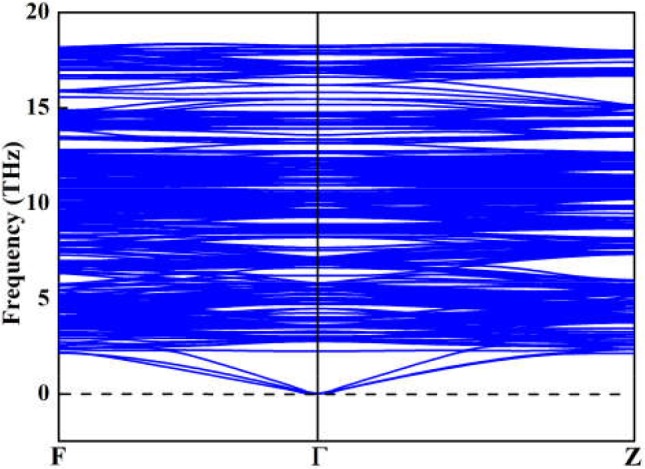
Simulated phonon dispersions of the lowest energy structure for Y_2_O_3_:Ce.

Although the X-ray powder diffraction (XRD) patterns of Y_2_O_3_:Ce crystals have been extensively studied, there appears to be inconsistencies of the spectra (Chien and Yu, 2008; Taibeche et al., 2016; Kumar et al., 2017). In order to clarify the crystal characters of the lowest-energy structure, we simulate the XRD patterns of Y_2_O_3_:Ce in the 2θ range of 15–65°. The result compared with experimental data is presented in Figure 3. It is evident that the calculated spectrum is in perfect agreement with the values measured by Kumar et al. (2017), demonstrating the validity of the lowest energy structure as well as the accuracy of our theoretical calculations. It should be pointed out that the simulated diffraction peak at 34° is ascribed to the (400) plane direction. This is accord with the result obtained by Taibeche et al. (2016) but different from the measured value proposed by Chien and Yu (2008). For comparison, the XRD patterns of the four isomers (a), (b), (c), and (d) are also provided in Figure 3. Although the overall distribution of the peaks in isomers is closely similar with each other, there are minor differences in the relative intensities. To evaluate the dynamical stability of Y_2_O_3_:Ce, the phonon spectrum within the Brillouin zone of ground state structure are calculated. Figure 4 illustrates the phonon dispersion curves along the high-symmetry directions including F, Γ, and Z. Clearly, the overall values in Figure 4 are positive and no virtual frequencies are observed in the full Brillouin zone. It is concluded that the rhombohedral phase structure of Y_2_O_3_:Ce crystal is dynamically stable.

### Electronic Properties

**Figure 5:**
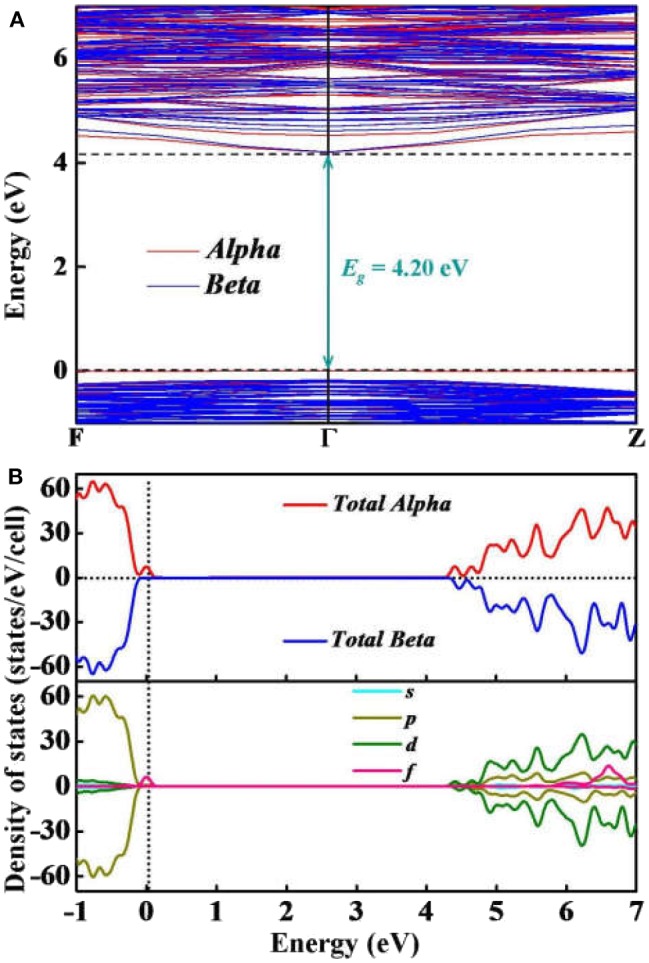
Simulated **(A)** electronic bands and **(B)** total and partial DOS of Y_2_O_3_:Ce.

To further elucidate the electronic properties of Y_2_O_3_:Ce crystal, we have performed a series of *ab initio* calculations including the electronic band structures, total and partial electronic density of states and electron localization functions. The calculated band structure and density of states (DOS) are plotted together in Figure 5. Our calculated results show that both of the conduction band minimum and valence band maximum are identified at Γ site. The band structure is considered to be a typical of semiconductor with relatively flat top of the valence bands. According to the calculations, the band gap value of ground state Y_2_O_3_:Ce is equal to 4.20 eV directly at Γ point. This result is very close to the energy gap of Y_2_O_3_:Nd system (Ju et al., 2020) but significantly smaller than that of pure Y_2_O_3_ crystal (Wilk and Wallace, 2002). The direct band gap of 4.20 eV suggests a semiconductor character of the Y_2_O_3_:Ce. In addition to the electronic band gap, the electronic calculations of high-symmetry directions are in accordance with the above analysis based on phonon spectrum. In Figure 5A, we can clearly see that the band structures can be divided into three parts. The high conduction band is above 4.20 eV while the low valence band is below −0.17 eV. Interestingly, an extremely narrow valence band is observed just below the Fermi level. This result is greatly different with the band structures of pure Y_2_O_3_. The calculations show that the narrow valence band is caused by the electronic Alpha states. In contrast, the Beta electrons are not identified near the Fermi level. In order to explore the origins of the electronic bands, we further calculate the partial DOS including s, p, d and f states. The calculated DOS are depicted in Figure 5B. It can be clearly seen that the high conduction bands are mainly formed by d and p states. The p electrons are calculated to be the strongest state in the low valence bands. Moreover, the partial DOS of Y_2_O_3_:Ce reveals that the extremely narrow valence band near Fermi level is ascribed to the f orbital, which suggests that the impurity Ce^3+^ ion leads to a dramatic reduction of the band gap. In other words, it is concluded that the incorporation of the doped Ce^3+^ ion into Y_2_O_3_ host crystal results in an insulator to semiconductor transition.

**Figure 6:**
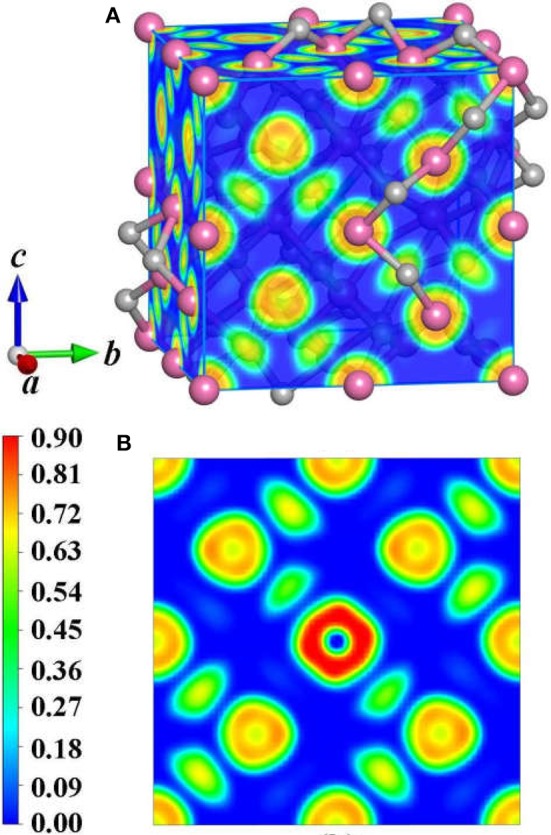
ELF of the **(A)** structure and **(B)** <100> plane for Y_2_O_3_:Ce.

**Figure 7:**
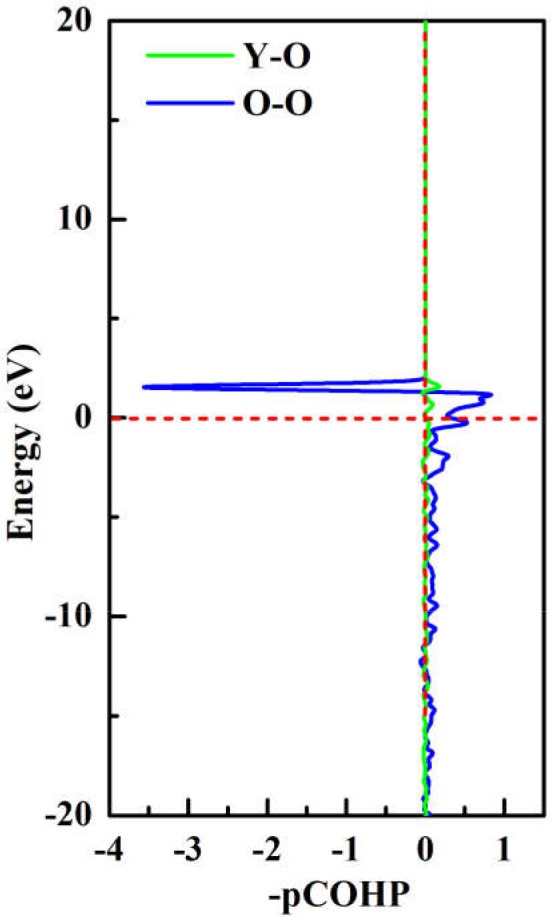
The projected Crystal Orbital Hamilton Population (-pCOHP) curves for the O-O bonds in Y_2_O_3_:Ce.

To achieve foundational understanding of the bonding character and distribution of electrons of Y_2_O_3_:Ce crystal, we have carried out a calculation on the electron localization functions based on the ground state structure. The visually ELF of the structure and (100) plane are presented together in Figure 6. Obviously, the electrons near the cationic atoms are greatly localized with ELF values at ~0.9 while the ELF values in the crystal lattice are nearly zero. This result indicates that the electrons localization on Ce and Y atoms broadens toward O atoms, forming a complete charge delocalization in the vicinity of O atoms. The strong ionic bonds are identified between Ce-O and Y-O. Furthermore, our calculations also show that the value of ELF at Ce atom is relatively larger than the ELF of Y atoms. This phenomenon can be explained as the remaining 4f (Masanori et al., 2020) electron of Ce^3+^ ion. It should be pointed out that there are strong charge localizations between O-O atoms, demonstrating the covalent bond of O atoms. To further quantitatively estimate the contribution of bonds between O atoms, we have presented the projected Crystal Orbital Hamilton Population (-pCOHP) curves for the O-O bonds in Y_2_O_3_:Ce. As shown in Figure 7, the strong bonding contributions of O-O bonds are evidenced. The bond features near the Fermi level can be ascribed to covalent. It is convinced that the excellent stability of Y_2_O_3_:Ce crystal is owing to the strong covalent bonds of O atoms.

## Conclusion

To summarize, we have systematically reported the structural evolutions, doping site locations and electronic properties of Y_2_O_3_ crystal doped with Ce^3+^ ions. By using the CALYPSO method in conjunction with first-principles calculations, a novel stable phase with R-3 space group is identified for the first time. For the ground state structure, the doped Ce^3+^ can naturally occupy the central Y^3+^ site in the crystal lattice of Y_2_O_3_, forming a standard cage-like structure. The cationic site symmetry of Ce^3+^ is calculated to be C_3i_ with six equal Ce–O bonds. The first four candidate isomers present different doping sites for Ce^3+^, which is helpful to investigate the structural evolution of Y_2_O_3_:Ce. By comparing the simulated XRD patterns with experimental data, we demonstrate the validity of the lowest energy structure. The dynamically stability of Y_2_O_3_:Ce crystal is carefully examined through the calculation of phonon dispersions. Our results of electronic band structures reveal that both of the conduction band minimum and valence band maximum are located at Γ site, leading to a band gap value of 4.20 eV. This band gap suggests a semiconductor character of Y_2_O_3_:Ce system. Interestingly, an extremely narrow valence band near Fermi level is observed in the band structure and the contribution of this band is assigned to f orbital. In addition, the calculated results of visually ELF show that the charge localizations between O-O atoms are dramatically strong, suggesting the covalent bond character of O atoms in the Y_2_O_3_:Ce crystal. These findings could provide important information of the microstructures of rare-earth doped laser materials.

## Data Availability Statement

All datasets generated for this study are included in the article/supplementary material.

## Author Contributions

MJ, JW, and CZ conceived the idea. MJ, JH, YJ, YC, WS, and SL performed the calculations. MJ, JH, and WS wrote the manuscript. All authors reviewed the manuscript.

## Conflict of Interest

The authors declare that the research was conducted in the absence of any commercial or financial relationships that could be construed as a potential conflict of interest.

## References

[B1] Atsushi T. Fumiyasu O. Isao T. (1993). First-principles calculations of the ferroelastic transition between rutile-type and CaCl_2_-type SiO_2_ at high pressures. Phys. Rev. B 78:134106.

[B2] Becke A. D. Edgecombe K. E. (1990). A simple measure of electron localization in atomic and molecular systems. J. Chem. Phys. 92, 5397–5403. 10.1063/1.458517

[B3] Chien W. C. Yu Y. Y. (2008). Preparation of Y_2_O_3_:Ce^3+^ phosphors by homogeneous precipitation inside bicontinuous cubic phase. Mater. Lett. 62, 4217–4219. 10.1016/j.matlet.2008.06.041

[B4] Gieszczyk W. Bilski P. Kłosowski M. Mrozik A. Zorenko Yu Zorenko T. (2019). Luminescent properties of undoped and Ce^3+^ doped crystals in Y_2_O_3_-Lu_2_O_3_-Al_2_O_3_ triple oxide system grown by micro-pulling-down method. Opt. Mater. 89, 408–413. 10.1016/j.optmat.2019.01.023

[B5] Han Z. K. Zhang L. Liu M. Maria V. G. P. Gao Y. (2019). The structure of oxygen vacancies in the near-surface of reduced CeO_2_ (111) under strain. Front. Chem. 7:436 10.3389/fchem.2019.00795 31275923 PMC6592146

[B6] Herbst J. F. Waston R. E. (1978). Relativistic calculations of 4f excitation energies in the rare-earth metals: further results. Phys. Rev. B 17, 3089–3098. 10.1103/PhysRevB.17.3089

[B7] Jia W. Wang Y. Fernandez F. Wang X. Huang S. Yen W. M. (2001). Photoluminescence of Ce^3+^,Tb^3+^:Y_2_O_3_ nanoclusters embedded in SiO_2_ sol-gel glasses. Mater. Sci. Eng. C 16, 55–58. 10.1016/S0928-4931(01)00298-3

[B8] Ju M. Lu C. Yeung Y. Kuang X. Wang J. Zhu Y. (2016). Structural evolutions and crystal field characterizations of Tm-doped YAlO_3_: new theoretical insights. ACS Appl. Mater. Interfaces 8, 30422–30429. 10.1021/acsami.6b09079 27734663

[B9] Ju M. Sun G. Kuang X. Lu C. Zhu Y. Yeung Y. (2017). Theoretical investigation of the electronic structure and luminescence properties for NdxY1–xAl_3_(BO_3_)4 non-linear laser crystal. J. Mater. Chem. C 5, 7174–7181. 10.1039/C7TC01911D

[B10] Ju M. Xiao Y. Sun W. Lu C. Yeung Y. (2020). In-depth determination of the microstructure and energy transition mechanism for Nd^3+^-doped yttrium oxide laser crystals. J. Phys. Chem. C 124, 2113–2119. 10.1021/acs.jpcc.9b10482

[B11] Ju M. Xiao Y. Zhong M. Sun W. Xia X. Yeung Y.. (2019b). New theoretical insights into the crystal-field splitting and transition mechanism for Nd^3+^-doped Y_3_Al_5_O_12_. ACS Appl. Mater. Interfaces 11, 10745–10750. 10.1021/acsami.9b00973 30789696

[B12] Ju M. Zhong M. Lu C. Yeung Y. (2019a). Deciphering the microstructure and energy-level splitting of Tm^3+^-doped yttrium aluminum garnet. Inorg. Chem. 58, 1058–1066. 10.1021/acs.inorgchem.8b02009 30216052

[B13] Kresse G. Furthmuller J. (1996). Efficient iterative schemes for ab initio totalenergy calculations using a plane-wave basis set. Phys. Rev. B 54, 11169–11186. 10.1103/PhysRevB.54.11169 9984901

[B14] Kresse G. Hafner J. (1993). Ab initio molecular dynamics for liquid metals. Phys. Rev. B 47, 558–561. 10.1103/PhysRevB.47.558 10004490

[B15] Krutikova I. Ivanov M. Murzakaev A. Nefedova K. (2020). Laser-synthesized Ce^3+^ and Pr^3+^ doped Y_2_O_3_ nanoparticles and their characteristics. Mater. Lett. 265:127435 10.1016/j.matlet.2020.127435

[B16] Kumar P. Nagpal K. Gupta B. K. (2017). Unclonable security codes designed from multicolour luminescent lanthanide doped Y_2_O_3_ nanorods for anti-counterfeiting. ACS Appl. Mater. Interfaces 9, 14301–14308. 10.1021/acsami.7b03353 28394563

[B17] Li L. Hao J. Liu H. Li Y. Ma Y. (2014). The metallization and superconductivity of dense hydrogen sulfide. J. Chem. Phys. 140:174712. 10.1063/1.4874158 24811660

[B18] Lin Y. C. Bettinelli M. Karlsson M. (2019). Unraveling the mechanisms of thermal quenching of luminescence in Ce^3+−^doped garnet phosphors. Chem. Mater. 31, 3851–3862. 10.1021/acs.chemmater.8b05300

[B19] Liu Y. Hu S. Zhang Y. Wang Z. Zhou G. Wang S. (2020). Crystal structure evolution and luminescence property of Ce^3+^-doped Y_2_O_3_-Al_2_O_3_-Sc_2_O_3_ ternary ceramics. J. Eur. Ceram. Soc. 40, 840–846. 10.1016/j.jeurceramsoc.2019.10.022

[B20] Loitongbam R. S. Singh W. R. Phaomei G. Singh N. S. (2013). Blue and green emission from Ce^3+^ and Tb^3+^ co-doped Y_2_O_3_ nanoparticles. J. Lumin. 140, 95–102. 10.1016/j.jlumin.2013.02.049

[B21] Lu C. Amsler M. Chen C. (2018). Unraveling the structure and bonding evolution of the newly discovered iron oxide FeO_2_. Phys. Rev. B 98:054102 10.1103/PhysRevB.98.054102

[B22] Lu C. Chen C. (2018). High-Pressure evolution of crystal bonding structures and properties of FeOOH. J. Phys. Chem. Lett. 9, 2181–2185. 10.1021/acs.jpclett.8b00947 29649871

[B23] Lu C. Li Q. Ma Y. Chen C. (2017). Extraordinary indentation strain stiffening produces superhard tungsten nitrides. Phys. Rev. Lett. 119:11550. 10.1103/PhysRevLett.119.115503 28949242

[B24] Lu C. Miao M. Ma Y. (2013). Structural evolution of carbon dioxide under high pressure. J. Am. Chem. Soc. 135, 14167–14171. 10.1021/ja404854x 24004352

[B25] Lupei A. Lupei V. Hau S. (2017). Vibronics in optical spectra of Yb^3+^ and Ce^3+^ in YAG and Y_2_O_3_ ceramics. Opt. Mater. 63, 143–152. 10.1016/j.optmat.2016.06.024

[B26] Marin R. Back M. Mazzucco N. Enrichi F. Frattini R. Benedetti A.. (2013). Unexpected optical activity of cerium in Y_2_O_3_:Ce^3+^,Yb^3+^, Er^3+^ up and down-conversion system. Dalton Trans. 42, 16837–16845. 10.1039/C3DT51297E 24085310

[B27] Masanori N. Akira M. Daisuke U. Yuki M. Yosuke G. Yoshikazu M. (2020). Flux growth and superconducting properties of (Ce,Pr)OBiS_2_ single crystals. Front. Chem. 8:44 10.3389/fchem.2020.00044 32117872 PMC7010857

[B28] Ming C. Pei M. Ren X. Xie N. Cai Y. Qin Y. (2018). Improving luminescent penetrability by Tm^3+^/Ce^3+^ doped Y_2_O_3_ nanocrystals for optical imaging. Mater. Lett. 218, 154–156. 10.1016/j.matlet.2018.01.169

[B29] Momma K. Izumi F. (2011). VESTA 3 for three-dimensional visualization of crystal, volumetric and morphology data. J. Appl. Crystallogr. 44, 1272–1276. 10.1107/S0021889811038970

[B30] Perdew J. P. Burke K. Ernzerhof M. (1996). Generalized gradient approximation made simple. Phys. Rev. Lett. 77, 3865–3868. 10.1103/PhysRevLett.77.3865 10062328

[B31] Richard D. Peter E. B. (1993). Crystal Orbital Hamilton Populations (COHP). Energy-resolved visualization of chemical bonding in solids based on density-functional calculations. J. Phys. Chem. 97, 8617–8624. 10.1021/j100135a014

[B32] Savin A. Jepsen O. Flad J. Andersen O. K. Preuss H. von Schnering H. G. (1992). Electron localization in solid-state structures of the elements: the diamond structure. Angew. Chem. Int. Ed. Engl. 31, 187–188. 10.1002/anie.199201871

[B33] Stefan M. Volker L. D. Andrei L. T. Richard D. (2016). LOBSTER: a tool to extract chemical bonding from plane-wave based DFT. J. Comput. Chem. 37, 1030–1035. 10.1002/jcc.24300 26914535 PMC5067632

[B34] Taibeche M. Guerbous L. Boukerika A. Kechouane M. Nedjar R. Zergoug T. (2016). Ab-initio simulations at the atomic scale of an exceptional experimental photoluminescence signal observed in Ce^3+^- doped Y_2_O_3_ sesquioxide system. Opt. 127, 10561–10568. 10.1016/j.ijleo.2016.08.092

[B35] Volker L. D. Andrei L. T. Richard D. (2011). Crystal Orbital Hamilton Population (COHP) analysis as projected from plane-wave basis sets. J. Phys. Chem. A 115, 5461–5466. 10.1021/jp202489s 21548594

[B36] Wang N. He J. Ye K. Song X. L T. (2018). Sol-gel synthesis and enhanced 1.54 μm emission in Y_2_O_3_:Yb^3+^, Er^3+^ nanophosphors co-doped with Ce^3+^ ions. Infrared Phys. Technol. 93, 77–80. 10.1016/j.infrared.2018.07.023

[B37] Wang Y. Lv J. Zhu L. Ma Y. (2010). Crystal structure prediction via particleswarm optimization. Phys. Rev. B 82:094116 10.1103/PhysRevB.82.094116

[B38] Wang Y. Lv J. Zhu L. Ma Y. (2012). CALYPSO: a method for crystal structure prediction. Comput. Phys. Commun. 183, 2063–2070. 10.1016/j.cpc.2012.05.008

[B39] Wilk G. Wallace R. M. (2002). Alternative gate dielectrics for microelectronics. Mater. Res. Soc. Bull. 27:3 10.1557/mrs2002.70

[B40] Zhu L. Wang X. Yu G. Hou X. Zhang G. Sun J. (2008). Effect of Ce^3+^ doping and calcination on the photoluminescence of ZrO_2_ (3% Y_2_O_3_) fibers. Mater. Res. Bull. 43, 1032–1037. 10.1016/j.materresbull.2007.04.025

